# A phase Ib study to assess the safety of the human papillomavirus DNA vaccine (AMV002) in combination with durvalumab for HPV-associated oropharyngeal squamous cell carcinoma

**DOI:** 10.3389/fonc.2024.1419258

**Published:** 2024-07-05

**Authors:** Rahul Ladwa, Janin Chandra, Wai-Ping Woo, Neil Finlayson, Howard Liu, Margaret McGrath, Adrienne See, Brett G. Hughes, Caroline L. Cooper, Jim E. Jackson, Marcin Dzienis, Yan Xu, Benedict Panizza, Ian Frazer, Sandro V. Porceddu

**Affiliations:** ^1^ Department of Cancer Services, Princess Alexandra Hospital, Brisbane, Australia; ^2^ Frazer Institute, Faculty of Medicine, The University of Queensland, Brisbane, Australia; ^3^ Jingang Medicine (Australia) Pty Ltd, Brisbane, Australia; ^4^ Department of Cancer Services, Royal Brisbane Hospital, Brisbane, Australia; ^5^ Pathology Queensland, Princess Alexandra Hospital, Brisbane, Australia; ^6^ Department of Cancer Services, Gold Coast University Hospital, Gold Coast, Australia; ^7^ ENT Department, Princess Alexandra Hospital, Brisbane, Australia

**Keywords:** immune checkpoint inhibitor, HPV - human papillomavirus, HPV vaccination, immunotherapies, oropharangeal cancer

## Abstract

**Background:**

Programmed cell death ligand 1 (PD-L1) inhibitors have limited efficacy as monotherapy in patients with recurrent/metastatic (R/M) Human Papilloma Virus (HPV) oropharyngeal squamous cell carcinoma (OPSCC). A phase I study of the therapeutic HPV-16 DNA vaccine AMV002 in curatively treated patients with OPSCC demonstrated a measurable immune response against HPV while being associated with high safety and tolerability. This prospective phase Ib single centre pilot study aims to test the safety and tolerability of combined PD-L1 inhibitor, Durvalumab, with AMV002 in 12 patients with recurrent OPSCC.

**Methods:**

Participants had evidence of R/M HPV-associated OPSCC. They received three intradermal administrations of AMV002 with Durvalumab followed by Durvalumab maintenance. Safety and tolerability data was the primary endpoint. The study was conducted with ethical approval (HREC/2018/QMS/47293) in Brisbane, Australia.

**Findings:**

The most common adverse event (AE) related to vaccine administration was erythema at the injection site. There were no grade 3 or 4 vaccine related AEs. There was one presumed immune-related grade 3 elevation in lipase secondary to Durvalumab with no intervention required. No patient ceased study due to treatment-related AEs. At week 16, objective response rate was 8% (N=1) and disease control rate was 17% (N=2). At a median follow up of 25.6 (20.0-26.6) months there was one long term complete response while all other participants developed progressive disease. Of the 11 evaluated patients, 9, (82%) had E6 and/or E7-specific T cell responses to the vaccine.

**Conclusion:**

The combination of AMV002 therapeutic HPV-16 vaccine and Durvalumab was found to be safe and well tolerated with no increased safety signals generated. T cell responses to vaccine were observed but further work will be required to improve efficacy.

## Introduction

The incidence of oropharyngeal squamous cell carcinoma (OPSCC) is rising globally, predominantly due to human papillomavirus (HPV) infection (1–3). In the USA, the annual incidence of HPV-associated OPSCC has increased by 225% since the 1980s. Unlike smoking-related OPSCC, locally advanced HPV-associated OPSCC has a good prognosis with treatment. However, the prognosis is poor in recurrent or metastatic (R/M) disease.

Treatment with immune checkpoint inhibitors (ICI) monotherapy targeting the Programmed cell death – protein/ligand 1 (PD-1/PD-L1) pathway in R/M mucosal head and neck SCC (HNSCC) has demonstrated a benefit over cytotoxic chemotherapy plus cetuximab most pronounced in tumours with higher levels of PD-L1 expression (3). The combination of antigen specific immunotherapy, which can induce a tumour-specific adaptive immune response, with ICI may be a further strategy to eliminate HPV-transformed advanced HNSCC and improve patient outcomes.

HPV-transformed cells express the viral oncoproteins E6 and E7 and can therefore be recognised and killed by cellular adaptive immune responses directed at these proteins (4). The combination of therapeutic vaccines inducing tumour-specific adaptive immunity and T cell targeted immunotherapy has the potential to increase response rates (5). Novel ICI combinations with therapeutic vaccines have been evaluated in Phase I/II studies of pre-treated R/M HPV-associated OPSCC. MEDI0457, a DNA vaccine of HPV-16 and HPV-18 E6/E7 expressing plasmids coupled with IL-12 expressing plasmids (6); ISA-101, consisting of HPV-16 E6 and E7 synthetic long peptides in combination with nivolumab (7, 8); and modified vaccine virus Ankara (MVA) vector expressing HPV16 E6/E7 and IL-2, TG4001 (tipapkinogene sovacivec) in combination with avelumab (9) have all shown a favourable safety profile.

We have developed a polynucleotide-based therapeutic HPV vaccine AMV002, comprised of plasmids encoding expression of two variants of a fusion protein of HPV16 E6 and E7. The AMV002 DNA vaccine is a 1:1 mixture of NTC8485-O-E6E7 and NTC8485-O-UE6E7 plasmids comprised of the NTC8485 expression vector minus the enhanced green fluorescent protein sequence to induce humoral immunity (10). Both plasmids encode a codon-optimized fusion protein of the HPV16 E6 and E7 viral sequences, which is additionally linked to ubiquitin in NTC8485-O-UE6E7, facilitating antigen processing and induction of cytotoxic T cells. The codon usage of the DNA encoding E6 and E7 in the AMV002 immunotherapy was optimised to maximise E7-specific humoral and cell-mediated immune responses (11) and control of E7-expressing tumours following intradermal administration in mice (10).

Pre-clinical evaluation has established that intradermal delivery of AMV002 in mice is safe and induces balanced humoral and cell-mediated immune responses (10). In a phase I, open-label, single centre dose escalation study of administration AMV002 in patients with curatively treated HPV-associated OPSCC, AMV002 was well tolerated at all dose levels and resulted in enhanced E6- or E7-specific cell mediated immunity to virus-derived tumour-associated antigens (12). The study indicated a therapeutic potential of AMV002 for treatment of HPV-associated malignant disease.

We conducted a phase Ib pilot study to assess the safety and tolerability of intradermal (ID) injection of the HPV-16 DNA vaccine (AMV002) when administered with Durvalumab (MEDI4736) for R/M HPV-related OPSCC.

## Methods

### Ethics statement

Subjects provided voluntary written informed consent. This clinical trial was approved by the Metro South Hospital and Health Service Human Research Ethics Committee (HREC/2018/QMS/47293) in Brisbane, Australia. The study was registered on the Australian New Zealand Clinical Trials Registry: ACTRN12620000406909. The study was subject to oversight by a Safety Monitoring Committee (SMC), which reviewed the toxicity data and all clinically relevant information.

### Study design and treatment

This Phase Ib, single centre, open label study of AMV002 co-administered with Durvalumab, evaluated the safety, tolerability and exploratory efficacy of the regimen in R/M HPV-related OPSCC. Twelve participants were enrolled; all participants received AMV002 and Durvalumab and were treated at the Princess Alexandra Hospital, Brisbane Australia.

AMV002 was given as three administrations, four weeks apart (Day 0, 28, 56) at a dose level of 1mg. It was administered via two intradermal injections for each administration, with one injection (containing up to a maximum of 0.5 mg AMV002) in each forearm. AMV002 consisted of a 1:1 mixture of the following plasmids - AMV002A: plasmid DNA NTC8485-O-E6E7 (encoding the secreted protein), and AMV002B: plasmid DNA NTC8485-O-UE6E7 (encoding the ubiquitin-tagged protein).

Durvalumab was initially administered using a weight based 10mg/kg dose by IV infusion on Day 7 and 28 followed on Day 56 with a fixed dose of 1500mg by IV infusion in the active treatment phase. Administration of Durvalumab was continued at a fixed dose of 1500mg by IV infusion in the maintenance treatment phase, with dosing every four weeks for 12 months, commencing four weeks after the final dose of AMV002. Study dosing in the maintenance treatment phase was discontinued in the event of disease progression, grade 3 or 4 toxicity, or patient refusal.

Radiological assessments were performed at baseline and at 16 weeks from start of trial and every 12-16 weeks thereafter. The investigator could choose to perform scans earlier, outside the protocol window, if they believed from clinical assessment that the patient’s disease was progressing and would warrant stoppage of treatment. Investigator assessed iRECIST was used to assess response at week 16. Adverse events were followed up to 12 months from date of first vaccine administration.

### Study population

Eligibility criteria for participants were as follows: (1) histologically or cytologically-confirmed R/M OPSCC or unknown primary of presumed OPSCC not amenable to definitive surgery and/or radiotherapy, (2) HPV-16 positivity (as confirmed by the presence of any positive test for HPV16 DNA or HPV16 mRNA or >70% p16 immunohistochemistry staining using CINtec^®^ Histology, Hoffmann-La Roche), (3) aged 18 years and older, (4) Eastern Cooperative Oncology Group (ECOG) performance status of 0-2 at enrolment, (5) patients with a life expectancy of >3 months.

Exclusion criteria were: (1) HNSCC of any other primary anatomic location in the head and neck, (2) received any prophylactic or therapeutic vaccine, or investigational drug, within 4 weeks of first vaccination, (3) history of severe allergy and reactions to any drugs (4) had received blood or plasma within 60 days prior to the screening visit, (5) receipt of the last dose of anticancer therapy within 28 days prior to the first dose of study drug, (6) use of immunosuppressive medication within 28 days before the first dose of study treatment, with the exceptions of intranasal, inhaled, injected or topical corticosteroids or systemic corticosteroids at physiological doses not to exceed 10 mg/day of prednisone, or an equivalent corticosteroid, (7) active or prior documented autoimmune or inflammatory disorders, (8) organ transplantation, (9) prior active malignancy, (10) brain metastases, (11) positive pregnancy test or active breastfeeding for female participants.

### Safety assessments

The incidence and severity of adverse events (AEs) were recorded and graded via CTCAE V4.03, from baseline to end of treatment phase, including vaccine-related AEs. Vaccine-related AEs included incidence and severity of local reactions at the vaccine injection site.

Treatment-emergent changes in vital signs (systolic and diastolic blood pressures, respiratory rate, pulse rate and aural temperature), clinical laboratory tests and physical examinations were recorded at specified intervals after each vaccination. Treatment-emergent AEs (TEAEs) were evaluated from the time of first dosing until day 84 follow-up visit.

Incidence of Serious Adverse Events (SAEs) within the study treatment period and up to 90 days after the last dose of therapy (excluding OPSCC changes) was recorded. AEs in the maintenance phase of durvalumab were collected, with clinical examination and laboratory assessments of full blood counts (FBC), biochemistry, amylase, lipase, and thyroid function tests performed at each treatment visit.

### Immunogenicity assessment

Change from baseline in peripheral T cell responses to HPV-16 E6 and E7 to end of active treatment phase (Day 84) were recorded, as measured by interferon gamma (IFN-γ) enzyme-linked immunosorbent spot (ELISPOT) assay (12) evaluated at baseline and on days 7, 35, 63 and 84 after the first vaccine dose. Stimulants were the immunodominant peptides for E6 (EVYDFAFRDL) and E7 (RAHYNIVTF), or pools of E6 or E7 overlapping peptides. A response to vaccination was defined as a ≥ 2-fold increase in spot count from baseline to at least one stimulation and at least one post-vaccination time point, and with a spot count of ≥ 20.

### PD-L1 IHC and interpretation

PD-L1 IHC diagnostic assays was performed on each archival specimen according to the manufacturer’s instructions: Ventana SP263 (rabbit monoclonal primary anti–PD-L1 antibody, prediluted, Ventana Medical Systems, Tucson, AZ). Interpretation of the SP263 assay was performed from stained slides by one of the authors (C.C.) who received appropriate training. PD-L1 expression in the tumor cell membrane and membrane and/or cytoplasm of tumor-associated mononuclear inflammatory cells such as lymphocytes and macrophages was scored. The combined proportional score (CPS) was defined as the total number of tumor cells and immune cells (including lymphocytes and macrophages) stained with PD-L1 divided by the number of all viable tumor cells, then multiplied by 100. Each countable array core section contained at least 100 viable tumour cells.

### Statistical analysis

The sample size was based on statistical modelling of previous clinical cancer phase I trials that have examined dose-toxicity and maximum tolerated dose, which usually ranges between 6-20 patients. This sample size of 12 was considered feasible, would provide sufficient evidence to assess the defined objectives and assist with design of a larger scale trial examining oncologic efficacy (13).

## Results

### Study cohort

Twelve subjects were enrolled over 12 months from 30^th^ July 2020 to 5^th^ July 2021. All patients were male, white Caucasian with a median age of 64 (50-78) years. Three participants were smokers and nine participants had a less than 5-pack year or absent smoking history. Eleven (92%) participants had received combined chemotherapy and radiation as part of their curative-intent therapy. All 12 patients had received prior radiotherapy with concurrent platinum chemotherapy (N=11) or cetuximab (N=1) for the primary disease. At recurrence, distribution was described as metastatic disease (lung N=5, Bone N=1) or locoregional (N=6). In the R/M setting patients had received up to two previous lines of therapy with four treatment naïve patients. Two patients received PD-1 inhibitor monotherapy as part of their systemic treatment and received a minimum of 3 cycles. PD-L1 CPS was evaluated as follows: PD-L1 >1 (range 2-60) N=6, PD-L1 <1 (N=3) and PD-L1 not evaluable N=3 due to inadequate tumour sample. Only two patients had a PD-L1 CPS >20. Patient demographics, PD-L1 assessment and treatment history are summarised in **Table 1**. Median follow up was 25.6 (20.0-26.6) months from registration.

**Table 1:** Baseline clinical characteristics of study population.

Clinical features		N
Age		64 (50-78)
Gender	Male	12
	Female	0
Smoking	Active	3
	Less than 5 Pack Years	3
	Never	6
Prior lines of therapy in R/M setting	Naive	4
	1	6
	2	2
Prior PD-1 therapy in R/M setting	Yes	2
	No	10
PD-L1 CPS status	<1%	3
	1-20%	4
	>20%	2
	Not evaluable	3
Recurrence	Metastatic	6
	Locoregional	6

### Safety and tolerability of AMV002 + durvalumab

All 12 patients completed 3 doses of vaccine administration. One patient completed the maximum planned 12 months of Durvalumab treatment. The median number of cycles of Durvalumab administration was six.

Treatment related AEs are summarized in **Table 2**. No patients withdrew from the study due to a treatment-related adverse event. There were no grade 3 or 4 adverse events related to the therapeutic vaccine AMV002. All study vaccine related AEs were mild in severity with the most common being acute erythema at the injection site occurring in all participants (N=12). One patient required prophylactic local anaesthetic cream prior to injections for injection-associated pain at the site. All AEs had resolved by date of study completion. There were no clinically significant alterations in haematology, biochemistry, coagulation, vital signs, or urinalysis reported for any participant. The most common Durvalumab related AE was rash, seen in four participants. There was one Grade 3 elevation of lipase which did not require any intervention. A Grade 3 colitis was deemed secondary to *Clostridium difficile* infection. One patient developed a new second primary cancer in the lateral tongue which was not p16 positive.

**Table 2:** Incidence and severity of Adverse Events.

	Adverse Events grade	Number of patients
Durvalumab related toxicity		
Skin Rash	2	2
Skin Rash	1	2
Constipation	1	1
Fatigue	1	1
Lethargy	1	1
Elevation of serum Lipase	3	1
Myalgia	1	1
Vaccine related toxicity		
Acute Erythema at injection site at time of administration	1	12
Pain at injection site	2	1

### Efficacy of AMV002 + durvalumab

At week 16, objective response rate was 8% (N=1) and disease control rate was 17% (N=2). Patients had progressive disease (N=10), stable disease (N=1) and partial response (N=1) as measured per immune related iRECIST. Ten patients discontinued treatment due to progressive disease, one ceased treatment due to becoming unwell with aspiration pneumonia, while one achieved a complete response at end of study visit following partial response at week 16. At follow-up of 12 months from dose of first vaccine administration, seven patients had died from their disease, four remained alive with disease, and one remained alive without active disease.

### Vaccine-induced cell-mediated immune responses

**Figure 1:**
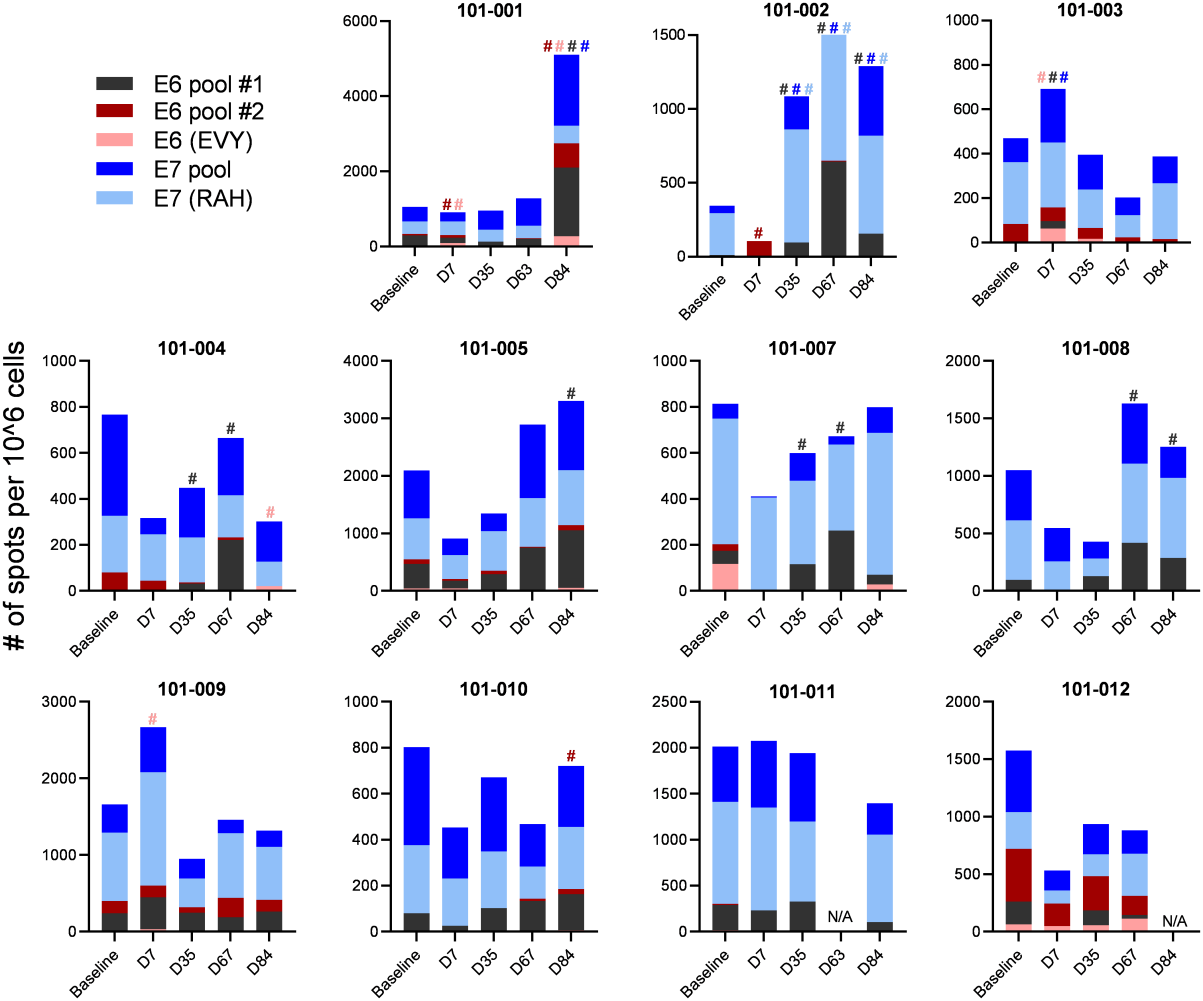
E6 and E7 specific T cell responses. Patient’s PBMCs were stimulated with pools of E6 and E7 overlapping peptides and assessed for IFNg secretion using ELISPOT. Spot numbers of media only controls were subtracted from samples stimulated with peptide. A response was defined as a minimal spot count of 20 and a 2-fold increase in spot number to any peptide pool at any time point compared to baseline spot numbers. A response is indicated by # with the corresponding colour to the peptide pool.

To evaluate E6 and E7 specific T cell responses, we assessed the interferon gamma (IFNg) secretion of patients PBMCs after stimulation with E6 and E7 peptides at baseline and at days 7, 35, 67 and 84 after the first vaccination using ELISPOT. A response was defined as a minimal 2-fold increase in spot numbers to any peptide pool at any time point compared to baseline. Of note, any spot counts below the count of 20 were deemed too low to include in this definition of response. Patient 101-006 yielded insufficient numbers of PBMCs at baseline and could not be evaluated for response. From the remaining 11 patients, all patients displayed pre-existing E6 and/or E7 specific T cell responses at baseline. After vaccination, 9 of 11 patients yielded an increased E6 and/or E7-specific T cell response (**Figure 1**).

## Discussion

This phase Ib study assessed safety and tolerability of intradermal (ID) injection of the HPV-16 DNA vaccine AMV002 when administered with Durvalumab (MEDI4736) for R/M HPV-associated OPSCC. There were no increased safety signals generated by combining the vaccine with the anti-PD-L1 agent durvalumab, either in terms of vaccine adverse effects or immune-related adverse events. The objective response rate in our study was 8% (N=1) which developed into a complete response at the end of study.

All evaluated 11 patients had pre-existing E6- and/or E7-specific T cell responses at baseline. According to our definition of response (at least 20 spots and at least a 2-fold increase of spot number to any peptide stimulation at any time point after vaccination and compared to baseline), we concluded that 9 of 11 evaluated patients had E6 and/or E7-specific T cell responses to the vaccine. Responses were observed to peptides where no response had been detected at baseline, and loss of responses was observed post vaccination that had been detected at baseline. We acknowledge that in the absence of placebo controls this study was not designed to assess vaccine immunogenicity in individual subjects.

HPV DNA vaccines that have entered clinical testing have been delivered intramuscularly via electroporation, often associated with pain, with a goal of enhancing immune responses (4). Intradermal administration may provide an alternate and more patient acceptable method for delivering DNA vaccines clinically. In a phase I, open-label, single centre dose escalation study of AMV002 in patients with definitively treated HPV-associated OPSCC, AMV002 was well tolerated at all dose levels and resulted in enhanced specific immunity to virus-derived tumour-associated antigens (12). Administration of up to 3 doses of 4mg of AMV002 was found to be safe and well tolerated, and E6- or E7-specific cell mediated immunity was observed in 10 of 12 subjects. The strength of the AMV002 vaccine technology is two-fold: (I) the E6E7 fusion protein sequence was codon-modified using a patented codon preference table (St. Lucia US 2011/0287039 A1), and (II), the vaccine consists of a 1:1 mixture of two plasmids (NTC8485- Os-E6E7 and NTC8485-O-UE6E7), one of them incorporating a ubiquitin repeat sequence to target the E6E7 fusion protein for proteasome degradation and antigen presentation for T cell activation, and the other plasmid incorporating a secretory sequence to induce antibody responses (REF PMID: 28166181).

MEDI0457 was studied in combination with anti-PDL1 durvalumab in a phase Ib/IIa trial in 35 patients with HPV-associated, ICI -naïve R/M HNSCC who had progressed on at least one prior regimen (14). Treatment-related AE were noted in 80% of patients, predominantly grade 1–2. Fatigue (37.1%) and injection site pain (34.3%) were the most common AEs. No patients had a grade 4/5 treatment-related AE. Objective response rate (ORR) was 27.6% [four complete responses, four partial responses]; responses were independent of PD-L1 tumor-cell expression (≥25% vs. <25%). HPV-16/18–specific T cells increased on treatment; 4 of 8 evaluable patients had a >2-fold increase in tumour-infiltrating CD8+ T cells. A phase II trial examined the combination of ISA-101 administered subcutaneously with nivolumab in patients in R/M HPV-16-positive cancers (8). Of the patients in this study, most (22 of 24), had advanced OPSCC. The most prevalent AEs in the study encompassed injection site reactions, fever, diarrhea, and hepatotoxicity. ORR was 33%. After vaccination, a variable increased number of HPV-specific T cells was observed in both responders and non-responders. The immune response did not correlate with any efficacy end points, suggesting that local factors in the tumour environment exert preeminent influences on vaccine effect. The modified vaccine virus Ankara (MVA) vector was evaluated in combination with avelumab in patients with R/M HPV-16+ cancers in a phase Ib/II trial (9). An interim analysis of nine patients (five of whom had OPSCC) showed no dose-limiting toxicities or serious adverse events and confirmed a partial clinical response in three patients. Of the five evaluable patients, at day 43 of immunization, three showed a detectable E6/E7-specific T cell response in the periphery, and four demonstrated an increase in CD8 infiltration and/or a decrease in infiltrated Treg/CD8 ratio in tissue (positive shift of CTL : Treg ratio). There appears to be consistency among the safety profile of the above published vaccine-directed trials in combination with ICI and the AE profile of this current study has raised no new safety concerns.

The most common response in this study was progressive disease with only one patient developing a PR at 16 weeks followed by complete response at the end of study visit. The study population was a heterogenous group with regards to PD-L1 status and prior systemic therapy which may have an impact on the observed efficacy of the therapeutic vaccine in combinations with Durvalumab in this study. Our study population was not restricted by PD-L1 status with 3 patients assessed as PD-L1 negative and only two patients had a PD-L1 CPS ≥20, considered favourable for benefit from ICI monotherapy. Two-thirds of the patient population had received prior systemic therapy, including two patients who had progressed on ICI. The efficacy was lower than that observed in the MEDI0457 which was an ICI naïve population with a high rate of PD-L1 ≥25% comprising 50% of evaluable cases (14). Unlike the study by (14), our inclusion criteria did not require histological confirmation of HPV status at R/M for enrolment. Despite this, studies have shown that HPV-related OPSCC retain HPV+/p16+ expression at recurrence. Future clinical studies of this treatment regime may benefit from targeting a patient population with early-stage OPSCC diagnosis, limited prior systemic treatments and a definite HPV16 genotyping and PD-L1 positivity status.

The combination of the therapeutic HPV-16 vaccine, AMV002, has been demonstrated to be safe and well -tolerated when administered to patients with R/M HPV-associated OPSCC. There were no increased safety signals generated by combining the vaccine with the anti-PD-1 agent durvalumab. T cell responses were induced to a polynucleotide vaccine delivered with ICI. Further investigative studies are warranted integrating cytokine induction and enhanced tumour specific T-cell responses.

## Data availability statement

The original contributions presented in the study are included in the article/supplementary material. Further inquiries can be directed to the corresponding author.

## Ethics statement

The studies involving humans were approved by Metro South Hospital and Health Service Human Research Ethics Committee (HREC/2018/QMS/47293) in Brisbane, Australia. The studies were conducted in accordance with the local legislation and institutional requirements. The participants provided their written informed consent to participate in this study.

## Author contributions

RL: Writing – original draft, Writing – review & editing. JC: Writing – original draft, Writing – review & editing. W-PW: Writing – review & editing. NF: Writing – review & editing. HL: Writing – original draft, Writing – review & editing. MM: Writing – original draft, Writing – review & editing. AS: Writing – review & editing. BH: Writing – original draft, Writing – review & editing. CC: Writing – original draft, Writing – review & editing. JJ: Writing – review & editing. MD: Writing – review & editing. YX: Writing – review & editing. BP: Writing – original draft, Writing – review & editing. IF: Writing – original draft, Writing – review & editing. SP: Writing – original draft, Writing – review & editing.
